# Atherosclerotic Plaque Stability Is Affected by the Chemokine CXCL10 in Both Mice and Humans

**DOI:** 10.4061/2011/936109

**Published:** 2011-11-13

**Authors:** Dolf Segers, Jonathan A. Lipton, Pieter J. M. Leenen, Caroline Cheng, Dennie Tempel, Gerard Pasterkamp, Frans L. Moll, Rini de Crom, Rob Krams

**Affiliations:** ^1^Department of Cardiology, Erasmus University Medical Center, 3522ZZ Rotterdam, The Netherlands; ^2^Department of Immunology, Erasmus University Medical Center, 3522ZZ Rotterdam, The Netherlands; ^3^Department of Cardiology, University Medical Center-Utrecht, 3584CX Utrecht, The Netherlands; ^4^Department of Vascular Surgery, University Medical Center-Utrecht, 3584CX Utrecht, The Netherlands; ^5^Department of Cell Biology and Genetics, Erasmus University Medical Center, 3522ZZ Rotterdam, The Netherlands; ^6^Department of Bioengineering, Royal School of Mines, Imperial College, London SW7 2AZ, UK

## Abstract

*Background*. The chemokine CXCL10 is specifically upregulated during experimental development of plaque with an unstable phenotype. In this study we evaluated the functional consequences of these findings in mice and humans. *Methods and Results*. In ApoE^−/−^ mice, we induced unstable plaque with using a flow-altering device around the carotid artery. From week 1 to 4, mice were injected with a neutralizing CXCL10 antibody. After 9 weeks, CXCL10 inhibition resulted in a more stable plaque phenotype: collagen increased by 58% (*P* = 0.002), smooth muscle cell content increased 2-fold (*P* = 0.03), while macrophage MHC class II expression decreased by 50% (*P* = 0.005). Also, the size of necrotic cores decreased by 41% (*P* = 0.01). In 106 human carotid endarterectomy specimens we found that increasing concentrations of CXCL10 strongly associate with an increase in atheromatous plaque phenotype (ANOVA, *P* = 0.003), with high macrophage, low smooth muscle cell, and low collagen content. *Conclusions*. In the present study we showed that CXCL10 is associated with the development of vulnerable plaque in human and mice. We conclude that CXCL10 might provide a new lead towards plaque-stabilizing therapy.

## 1. Introduction

Atherosclerosis is a progressive inflammatory disease of the arterial vasculature with lesions that may turn unstable, which may lead to lesion rupture. The high mortality of these ruptures has been associated with atherosclerotic plaques that display a specific histological phenotype, characterized by a large pool of lipids and necrotic cell debris covered by a thin fibrous cap and the presence of inflammatory cells in the plaque shoulders [1]. 

Previously, we developed an experimental animal model in which plaques with stable and unstable characteristics are simultaneously induced in a single straight arterial segment [2]. We subsequently identified specific expression profiles of various chemokine genes in vulnerable plaque development [3]. In these, the chemokine CXCL10 was specifically upregulated during the early phase in the development of the vulnerable plaque segment.

CXCL10 is a member of the CXC family of chemokines. Upon stimulation with interferon-gamma (IFN-*γ*) it is expressed by many cell types, for example, monocytes/macrophages, endothelial cells, fibroblasts, and natural killer cells [4, 5]. Effects of CXCL10 are mediated by the G-protein coupled receptor CXCR3, which is known to be expressed by resting and activated T cells, NK cells, and a subset of peripheral blood monocytes [5–7]. Furthermore, CXCL10 plays a variety of roles in inflammatory diseases, like in the initiation and maintenance of T-helper-1- (Th1-) polarized immune responses [8, 9], migration of activated T cells [10], but also of natural killer cells [11], of monocytes [12, 13] and of smooth muscle cells (SMC) [14].

Despite these compelling findings described in the literature, in atherosclerosis CXCL10 has been less completely characterized. In human atherosclerosis, but not in healthy arteries, endothelial cells, smooth muscle cells, and macrophages express CXCL10 [15]. Functional observations in CXCL10 gene knockout mice indicated that CXCL10 exerts pro-atherogenic effects, probably related to the specific recruitment and retention of activated Th1 cells and downregulation of a regulatory T-cell response [16]. While these findings indicate that CXCL10 plays a role in atherogenesis, its effect on plaque composition is unclear. Based on our previous observations on gene expression in the mouse model, we hypothesized that CXCL10 mediates development of vulnerable atherosclerotic plaque. Hence, we assessed whether CXCL10 plays a critical role in unstable plaque development by evaluating the effect of antibody-mediated functional inhibition of CXCL10 on lesion phenotype in our vulnerable plaque mouse model. To provide evidence that the findings in the mouse bear relevance for human atherosclerosis, we also investigated the association between CXCL10 concentrations and plaque composition in human carotid endarterectomy lesions.

## 2. Materials and Methods

### 2.1. Animals and Surgical Procedures

Apolipoprotein-E deficient mice, 15–20 weeks of age, were obtained from the Jackson Laboratories (Bar Harbor, ME, USA). They were fed a high fat, high cholesterol diet consisting of 15% (wt/wt) cocoa butter and 0.25% cholesterol (wt/wt) (Diet W, Hope Farms, Woerden, The Netherlands). Two weeks after this diet was initiated, the mice were instrumented with a shear stress-altering device around the right common carotid artery under 2% isoflurane anesthesia, as described previously [2]. Briefly, this device gradually narrows the vessel lumen to ~70% of its original diameter. As a consequence, shear stress is lowered upstream of the device, gradually increases inside the device, and is oscillating just downstream of the device [17]. After surgery, the mice were kept on the high fat, high cholesterol diet for the remainder of the experiment. The device remained in situ for 9 weeks. Intervention and control groups consisted of at least 10 animals per group. All animal experiments were approved by the institutional animal ethical committee and performed in compliance with institutional and national guidelines.

### 2.2. CXCL10 Inhibition

Mice were intravenously injected three times a week in week 2–4 after surgery with an anti-mouse bioactivity-neutralizing monoclonal CXCL10 antibody (MAB466, R&D Systems, Abingdon, United Kingdom) in a dose of 25 *μ*g in 0.2 mL sterile PBS. Untreated cast-instrumented mice served as control. 

### 2.3. Isolation and Analysis of Murine Tissues

Following sacrifice, blood was collected and mice were flushed systemically with PBS. Next, the common carotid artery was isolated, embedded in OCT compound (Tissue-Tek, Sakura, Japan), and snap-frozen in liquid nitrogen. For histology, 8 *μ*m cryosections were cut and stained for routine histology (Hematoxylin-Eosin, HE), lipids (Oil Red O), collagen (Picrosirius Red), macrophages (anti-CD68, AbD Serotec, Oxford, UK), macrophage activation (anti-Major Histocompatibility Complex (MHC) class II, clone M5/114, ATCC TIB-120), and smooth muscle cells (anti-SMC *α*-actin, Sigma-Aldrich, The Netherlands). Subsequently, high-resolution images were taken using an Olympus BX-40 microscope or a Zeiss LSM5 Meta confocal microscope (Zeiss Jena, Germany). Collagen stainings were assessed using crossed circular polarization filters. To calculate lesion size and cellular content relative to the plaque area, all images were analyzed after digitalization by automated image analysis software (Clemex Technologies Inc, Canada) applying thresholding of the stained areas. Necrotic cores (defined as hypocellular plaque cavities devoid of collagen, containing cholesterol clefts) were assessed based on HE and Picrosirius Red stained sections and measured relative to the lesion area. Total cholesterol levels were measured in serum samples by an enzymatic colorimetric method (Cholesterol E, Wako Diagnostics, USA).

### 2.4. Endarterectomy Procedures

All patients in this study were participants of a prospective study aimed at investigating the predictive value of plaque characteristics for long-term outcome (Athero-Express), of which the study methods have been described extensively before [18]. In short, all patients underwent carotid endarterectomy, during which arterial wall tissue was obtained. Next, the culprit lesion, defined as the segment with the largest plaque burden, was processed for histological examination. In addition to HE staining, sections were stained for collagen (Picrosirius Red), SMC (*α*-actin), and macrophage (CD68) content. Histological assessment was performed in a semiquantitative fashion (none, minor, moderate, heavy), on basis of two independent observers. When results contradicted, a third independent observer was consulted. Subsequently, plaques were designated as fibrous (lipids constitute <10% of lesion surface area), fibroatheromatous (10–40% lipids), or atheromatous (>40% lipids) based on collagen and HE stainings. Besides, plaques were also designated as SMC dominant or macrophage dominant based on the prevailing cell type obtained from the specific stainings. 

A 5 mm segment directly adjacent to the culprit lesion was crushed in liquid nitrogen, and subsequently total protein was extracted using 1 mL of TriPure Isolation Reagent (Boehringer Mannheim, Germany), according to the manufacturer's protocol. Then, CXCL10 concentration was measured by Enzyme-Linked Immuno Sorbent Assay (ELISA) according to manufacturers instructions (Quantikine human CXCL10 immunoassay, R&D Systems, Abingdon, United Kingdom) and blinded from histological and clinical data.

### 2.5. Statistical Analysis

SPSS (version 15.0, SPSS Inc., USA) was used for all analyses. To test the differences between the treated and the control mice at 9 weeks of cast placement, a student's *t*-test was used. When these data had a non-Gaussian distribution, a nonparametric *t*-test was used. For the endarterectomy samples, to compare CXCL10 levels between categorical baseline and plaque morphology variables, Kruskal-Wallis and Mann-Whitney tests for non-Gaussian distributed data were used. To investigate the differences between continuous baseline variables and CXCL10, patients were categorized into quartiles, named 1 through 4 based on increasing CXCL10 concentration. Subsequently, these quartiles were used to sort all other variables. One-way ANOVA was used to compare continuous variables. The quartiles were also used to visualize the relationship between CXCL10 concentration and plaque morphology. Data are described as mean ± SD or median (Inter Quartile Range) as appropriate. A *P* value of <0.05 was considered statistically significant.

The authors had full access to the data and take responsibility for its integrity. All authors have read and agree to the manuscript as written.

## 3. Results

### 3.1. Suppression of CXCL10 Bioactivity Inhibits Experimental Vulnerable Plaque Formation

**Figure 1:**
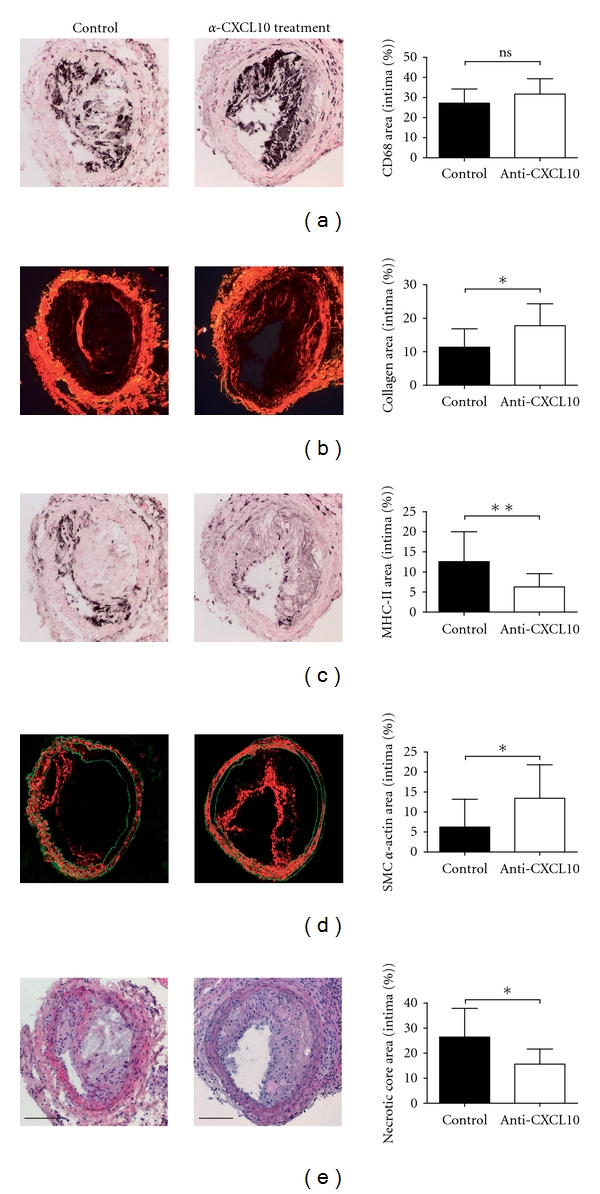
Anti-CXCL10 treatment in atherosclerosis susceptible mice results in a change into a more stable lesion phenotype. A flow-altering device around the common carotid artery induced atherosclerosis in ApoE^−/−^ mice. From week 1 to 4 of lesion development, a bioactivity-neutralizing anti-CXCL10 antibody was injected. After 9 weeks, lesions were compared to untreated controls by histology. The pictures show representative histological sections of treated and control mice. All photographs have been made with the same magnification (100x). Scale bars are provided in (e) and represent 100 *μ*m. Data in bar diagrams are the mean values ± SD of at least 8 sections from at least 10 different animals per group. CXCL10 inhibition resulted in a more stable morphology evidenced by unchanged amounts of lesion macrophages (a), increased amounts of collagen (b), decreased macrophage activation (c), increased numbers of SMC (d), and reduced necrotic core size (e). *P < 0.05, **P < 0.01. MHC-II: Major Histocompatibility Complex Class II, SMC: smooth muscle cell.

As we previously found that CXCL10 is expressed specifically in developing unstable lesions [3], we investigated if CXCL10 is also functionally involved in the development of plaque vulnerability using a mouse model that we developed before [2]. ApoE-deficient mice, in which a shear stress-altering device was applied, were injected with a bioactivity-neutralizing antibody during the onset of plaque formation. As expected, serum total cholesterol levels did not differ between treated and control mice (30.13 ± 4.6 versus 29.92 ± 6.7 mmol/L, *P* = 0.92). Short-term inhibition of CXCL10 did not influence the extent of plaque development, since we found no difference in lesion size between the treated and the control mice after 9 weeks of shear stress alteration. Because macrophage foam cells are characteristic of atherosclerosis, we measured both plaque lipid (31.3 ± 8.0% treated versus 29.5 ± 7.0% control) and macrophage content (31.7 ± 7.6% treated versus 27.8 ± 7.0 control; Figure 1(a)), where both remained unchanged upon CXCL10 inhibition.

To assess plaque vulnerability, we determined the amount of collagen in the lesions, which is the main stabilizing component of the plaque. Interestingly, we found a 57% increase in the relative amount of collagen in the plaques following CXCL10 suppression (17.8 ± 6.5% versus 11.3 ± 5.5%, *P* = 0.002; Figure 1(b)). The amount of plaque collagen is essentially the result of a balance between collagen deposition and breakdown. Therefore, the increase in collagen may be the result of decreased breakdown predominantly by proteinases secreted by activated macrophages. To determine the extent of immune activation, we measured MHC class II by immunohistochemistry. The cellular morphology, location in the plaque and spatial association of MHC class II staining with macrophage staining by CD68 antibodies in adjacent sections (Figures 1(a) and 1(c)) strongly suggests that MHCII-positive cells are the prime cells expressing this activation marker. We found a 50% reduction in the plaque MHC class II levels following CXCL10 inhibition (6.3 ± 3.3% versus 12.6 ± 7.4%, *P* = 0.005; Figure 1(c)). In addition, the amount of SMC, which is known to produce collagen, nearly doubled in the CXCL10-suppressed group (13.5 ± 8.4% versus 6.3 ± 7.0%, *P* = 0.03; Figure 1(d)), suggesting that the differences in collagen content may be explained by several factors.

The necrotic core is a hallmark component of the vulnerable plaque. To test whether CXCL10 inhibition reduces necrotic core formation, we analyzed both the number of necrotic cores in the lesions as well as their relative size. We found that CXCL10 inhibition resulted in fewer necrotic cores: 38.9 ± 22.1% versus 57.7 ± 20% of the sections covering the entire lesion that contained a necrotic core (*P* = 0.02). Moreover, also the relative size of the necrotic cores decreased following antibody treatment from 26.4 ± 11.4% to 15.6 ± 6.1% of the plaque surface area (*P* = 0.01; Figure 1(e)).

### 3.2. Patient Characteristics

**Figure 2:**
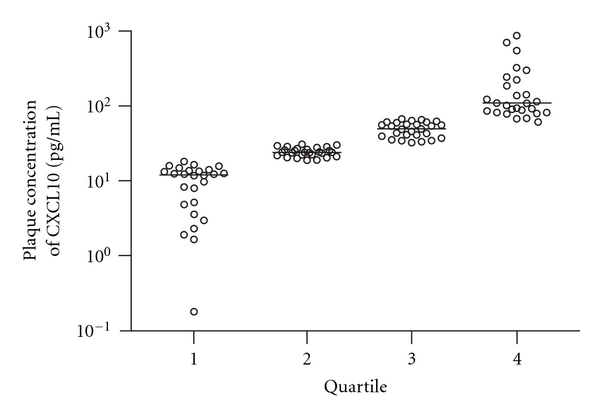
Distribution of CXCL10 measurements from each patient across quartiles. Atherosclerotic plaques were obtained from 106 patients during carotid endarterectomy. In the plaque segment directly adjacent to the culprit lesion, the content of the chemokine CXCL10 was measured by ELISA. Based on these measurements, patients were divided into one of four quartiles according to the CXCL10 concentration. Quartile 1 represents the lesions with the lowest CXCL10 values, whereas quartile 4 contains the highest. This figure shows the individual measurements for all patients and the distribution across each quartile of plaque CXCL10. Horizontal bars represent the median concentration of each quartile.

For this study endarterectomy specimens of 106 patients were analyzed. An overview of the patient characteristics is provided in Table 1. Histological examples of the lesions are shown in a previous publication by Verhoeven et al. [18]. The CXCL10 concentration in the specimens ranged from undetectable to 384.8 pg/mL, with a median (interquartile range) of 38.34 pg/mL (14–39 pg/mL). To compare continuous CXCL10 levels to the categorical variables, patients were categorized into quartiles (Figure 2). The variables were then tested for changes across the quartiles. No differences were found comparing risk factors for atherosclerotic disease. The use of medication did not differ significantly between the quartiles. 

### 3.3. High CXCL10 Concentrations Identify Patients with a More Vulnerable Plaque Phenotype

Several significant associations were found between plaque composition and CXCL10 levels. Atheromatous lesions were more prominent at higher CXCL10 concentrations than those classified as fibrous (Rank 53 versus 45, *P* = 0.003). Also, higher CXCL10 concentrations were associated with more macrophage-dominant plaques (Rank 56 versus 41, *P* = 0.009), fewer smooth muscle cells (Rank 39 versus 74, *P* = 0.001), and less collagen (Rank 37 versus 63, *P* = 0.003).

**Figure 3:**
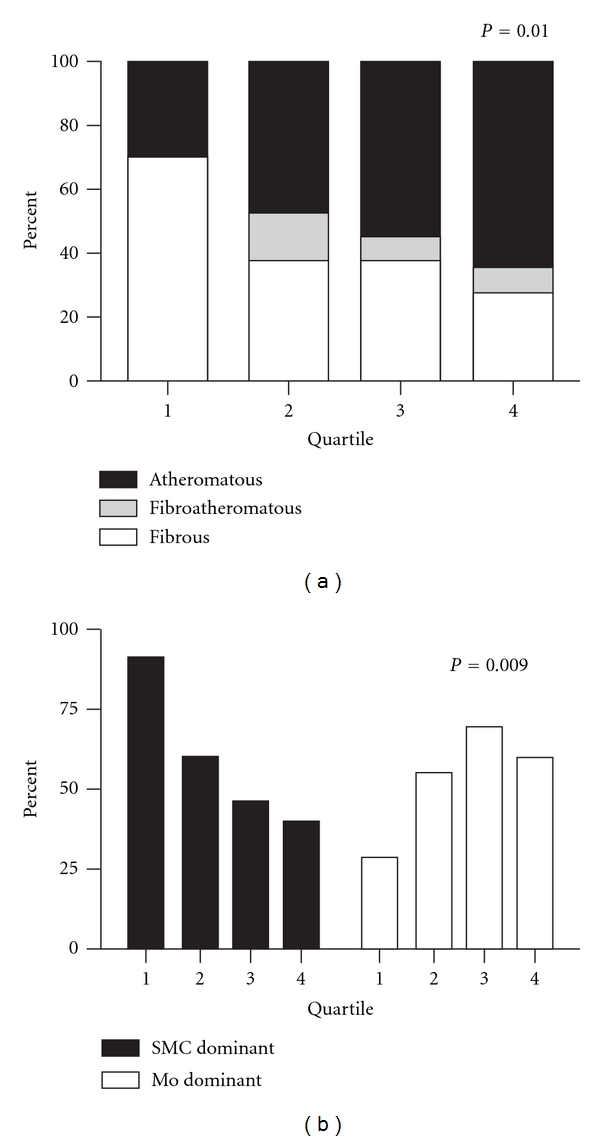
Plaque concentrations of CXCL10 are associated with lesion morphology. Individual values were divided over quartiles (see Figure 1). By using ANOVA, we tested to see if plaque CXCL10 concentrations associated with lesion morphology. Increasing plaque concentrations of CXCL10 were indeed associated with an increase in atheromatous-type lesions (a) and a decrease of SMC dominant lesions and an increase of macrophage dominant lesions (b).

**Figure 4:**
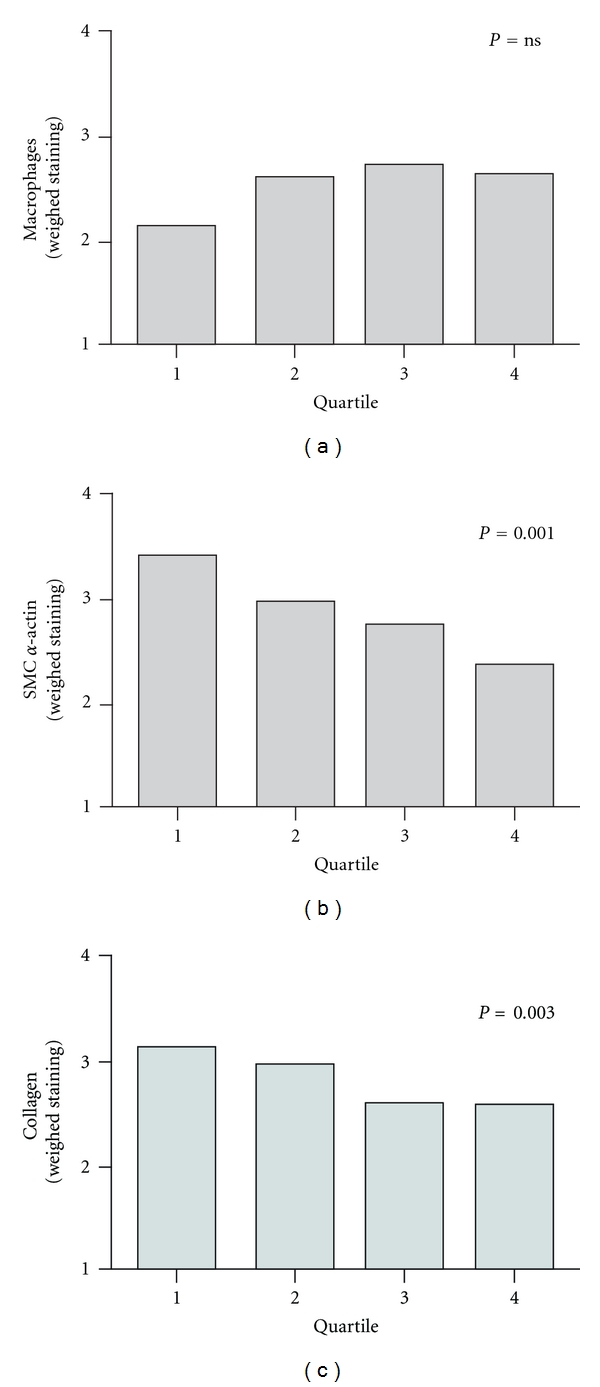
Increasing carotid plaque CXCL10 concentrations are associated with a less fibrous phenotype. Plaque concentrations of CXCL10 were associated with histological qualities of the lesion by ANOVA. Staining for macrophages, SMC, or collagen was quantified as none, minor, moderate, or high. Based on these qualifications a weighed value for average staining intensity was calculated (1, none; 2, minor; 3, moderate; 4, heavy staining). Macrophage staining was not associated with plaque CXCL10 (a), whereas high plaque concentrations of CXCL10 were associated with decreasing amounts of smooth muscle cells (SMC) (b), and decreasing collagen (c).

Subsequently, we analyzed the association between plaque composition and CXCL10 levels, by dividing patients into quartiles based upon CXCL10 concentration. More atheromatous lesions were represented in the higher quartiles, while fibrous lesions were more frequently present in the lower quartiles (Figure 3(a)). Macrophage-dominant lesions were mostly found in the top quartiles of CXCL10 content, while SMC dominance associated with lower CXCL10 concentrations (Figure 3(b)). However, no differences were seen in the macrophage staining between the quartiles (Figure 4(a)). This paradox was the result of an inverse relation between smooth muscle cell staining and increasing CXCL10 concentrations (Figure 4(b)). Additionally, more collagen staining was seen in the plaques in the lower quartiles (Figure 4(c)).

## 4. Discussion

This study may be summarized by its two main findings. First, we showed a functional role of CXCL10 in vulnerable plaque formation in an experimental mouse model for unstable atherosclerotic disease. We observed that inhibiting CXCL10 with a bioactivity-neutralizing antibody resulted in plaques with less macrophage activation, with more SMC and a subsequent increase in collagen content compared to controls. A decreased number and size of necrotic cores in the lesions of the treated animals further substantiated these findings. This resulted in a lesion phenotype with increased intrinsic stability.

Second, we found that in human carotid plaques, CXCL10 levels are associated with lesion morphology. This is evidenced by an increased number of atheromatous plaques with increasing CXCL10 concentrations, and by the association with unstable plaque characteristics, such as macrophage dominance and reduced presence of smooth muscle cells and collagen.

Previous studies have shown that inhibition of CXCL10 by antibodies in an intestinal inflammation animal model is an effective way to inhibit its function [19, 20]. The findings in those studies have been ascribed to the reduced amount of Th1 cells that were recruited to the diseased sites and either reduced production of inflammatory cytokines [19] or resulted in inflammatory disease exacerbation [20], depending on the role of T cells in the pathogenesis of the disease.

The lesion-stabilizing effect we observed by inhibiting CXCL10 might be explained by a similar mechanism as described above, as it has been shown before that inhibition of the CXCL10-CXCR3 pathway decreases the amount of lesional T cells [16, 21]. Veillard et al. reported that genetic deletion of CXCR3 in ApoE knockout mice reduced early lesion formation, which correlated with an increase in lesional FoxP3-positive regulatory T cells [21]. Recently, it has been reported that genetic deletion of CXCL10 in atherosclerosis susceptible mice also resulted in smaller lesions, which contained fewer CD4+ T cells [16]. Surprisingly, despite the decrease in overall T-cell presence in the lesions, a higher expression of FoxP3 as well as of the anti-inflammatory cytokines IL-10 and TGF-*β* mRNA was found. This suggests a more prominent role for regulatory T cells in this model. A similar increase in FoxP3 and TGF-*β* expression was found in a study using a peptide antagonizing CXCR3 in LDL receptor knockout mice, resulting in smaller lesions in the aortic root and the aorta [22]. 

A general limitation of our experimental mouse model is that there are few T cells in the induced lesions after 9 weeks of cast placement, approximately 2–4 per lesion cross section [3]. As a consequence, we have been unable to find a difference in T-cell numbers following CXCL10 inhibition (data not shown). Nevertheless, the reduction in T cells might have taken place during the early phase of actual inhibition. We performed measurements at only one time point, while the presence of T cells in a lesion changes over time [23]. Also, the effect of small numbers of T cells might prove to be very strong [24]. 

The small numbers of T cells do not allow to produce sufficiently reliable data to enable making a comparison with the situation in mice that are deficient in CXCL 10 by gene targeting. This cannot be solved by mRNA analyses, as there was no RNA available from the endarterectomy samples that have been used. Besides facilitating recruitment of leukocytes, CXCL10 also induces and sustains a dominant Th1-type response by increasing the production of IFN-*γ*, providing a positive feedback loop [25]. Neutralization of CXCL10 might therefore result in a decreased concentration of IFN-*γ* in the lesions, which is considered a key cytokine in disease progression [26] and macrophage activation [27]. This notion is in agreement with our findings regarding macrophage activation status as measured by MHC class II expression [27], SMC presence [27], and collagen accumulation [28]. 

An alternative or additional mechanism of plaque modification by CXCL10 inhibition might be operative as well. Not only Th1 and NK cells, as major IFN-*γ* producers, are responsive to CXCL10, but also plasmacytoid dendritic cells (PDC) express CXCR3 and are recruited via this receptor [29]. The numbers of circulating PDC are reduced in human atherosclerosis, while immunohistochemical analysis has indicated that they are recruited to advanced plaques [30, 31]. PDC are known to produce large quantities of IFN-*α* upon activation, and this cytokine functions as an inflammatory amplifier in the atherosclerotic plaque [32]. Additionally, a minor subset of monocytes has been shown to be recruited via CXCR3 to inflammatory environments [7] and might thus contribute to plaque instability as well.

Further research is needed to understand the mechanisms underlying the effects of CXCL10 inhibition. For example, apoptosis could have an important role in the observed differences in plaque composition and size. It could be of interest to study the role of SMC in more detail by staining serial sections for proliferation and apoptosis on the one hand and alpha actin on the other hand. In addition, in situ zymography studies could be performed to get a clearer idea of the possible role of collagenolytic activity in the lesions. It would be very interesting to know more about the role of the proinflammatory cytokine microenvironment in the plaque in the experiments with CXCL 10 inhibition. From the present data, we cannot exclude that by inhibiting CXCL10 we just postponed the process of the lesion formation and maturation rather than permanently altering it. For this argument and because of the ongoing discussion on the quality of animal models representing human disease states, we also performed a parallel study in humans.

Mach et al. [15] were the first to associate CXCL10 with human atherosclerosis, reporting that CXCL10 was expressed in several stages of the disease. CXCL10 was shown to be present on the surface of endothelial cells, suggesting a role in the recruitment and adhesion of CXCR3 positive cells from the circulation. They also showed broad expression of CXCL10 by SMC and macrophages throughout the lesion. Little is known about the effects of CXCL10 on human atherosclerosis development and clinical outcome. Previous studies sought to correlate plasma CXCL10 levels with the occurrence of coronary heart disease (CHD). In a case-control study it was found that CHD risk was associated with an increase in serum CXCL10 [33]. A later prospective study showed that indeed increased levels of CXCL10 exist prior to CHD, but was not considered an independent risk factor [34]. Our study is the first one to correlate lesional CXCL10 protein content with plaque morphology in humans and to be a possible predictor of plaque vulnerability. Obviously, this cannot be measured directly in living patients, so it would be of interest to know the correlation between lesional and systemic levels of CXCL10. There were no plasma samples available to measure the systemic CXCL 10 levels and correlate them to the lesional levels, so this has to await future work.

In conclusion, we showed that the chemokine CXCL10 plays a functional role in the destabilization of atherosclerotic plaques in mice and is specifically upregulated in vulnerable plaques in humans. Since the experimental data were paralleled by similar findings in human unstable lesions, CXCL10 might be regarded as a new lead into understanding the process of plaque destabilization. 

## Figures and Tables

**Table 1:** Patient baseline measurements for carotid endarterectomy specimens.

Clinical characteristics	Total	Quartile of CXCL10 concentration				*P* value*
		1	2	3	4	
CXCL10 pg/mL	38.3 (14–40)^ †^	0–11.3	11.8–18.4	19.3–37.2	37.6–382.6	0.000
Age (years)^†^	68.4 ± 8.9	66.0 ± 8.0	68.3 ± 10.2	67.7 ± 8.2	71.2 ± 8.5	0.157
Male (%)	73.6	73.1	70.4	66.7	84.6	0.316
Hypertension (%)	71.7	80.0	70.4	66.7	79.2	0.764
Smoker (%)	24.5	30.8	18.5	32.0	19.2	0.476
Symptomatic disease (%)	86.8	84.6	85.2	92.6	84.6	0.762
Statin (%)	56.6	66.7	55.6	55.6	56.0	0.530
*β* blocker (%)	39.6	37.5	33.3	56.0	41.7	0.321
ACE inhibitor (%)	39.6	42.3	33.3	40.7	42.3	0.881

Serum values^†^						
Total cholesterol (mmol/L)	4.9 ± 1.3	4.8 ± 0.9	5.2 ± 1.3	4.8 ± 1.7	4.6 ± 1.2	0.564
HDL (mmol/L)	1.2 ± 0.4	1.2 ± 0.4	1.4 ± 0.3	1.1 ± 0.3	1.1 ± 0.4	0.156
LDL (mmol/L)	3.0 ± 1.1	2.7 ± 0.8	3.3 ± 1.0	3.2 ± 1.4	2.7 ± 1.0	0.392
Triglycerides (mmol/L)	1.9 ± 0.9	2.3 ± 1.0	1.8 ± 0.8	2.0 ± 1.1	1.7 ± 0.7	0.434
ApoB (mmol/L)	0.9 ± 0.2	0.9 ± 0.2	1.0 ± 0.2	1.0 ± 0.3	0.9 ± 0.3	0.787
Glucose (mmol/L)	6.6 ± 1.9	5.8 ± 0.7	7.0 ± 2.0	7.0 ± 2.3	6.4 ± 1.9	0.129
CRP (mg/mL)	3.40	3.13	2.96	4.81	4.52	0.221
	(1.6–7.3)	(1.4–5.8)	(1.8–5.8)	(1.7–9.1)	(1.6–7.3)	

Baseline data of 106 patients were stratified into four quartiles according to plaque CXCL10 concentration. ^†^Values are represented as mean ± SD or median (Inter Quartile Range) as appropriate. *P values based on ANOVA test between CXCL10 quartiles for continuous variables and nonparametric Mann-Whitney test for categorical variables.
